# Rare Presentations of Sarcoidosis: A Case Series

**DOI:** 10.7759/cureus.37208

**Published:** 2023-04-06

**Authors:** Sangita D Kamath, Ajatshatru Upadhyay, Sreedevi Jakka

**Affiliations:** 1 Internal Medicine, Tata Main Hospital, Jamshedpur, IND; 2 Pathology, Tata Main Hospital, Jamshedpur, IND

**Keywords:** tuberculosis, sarcoid, meningitis, lymphadenopathy, arthralgias

## Abstract

Sarcoidosis is a systemic disease of unknown etiology with multi-system affection. It typically involves the skin, eyes, hilar lymph nodes, and pulmonary parenchyma. However, as any organ system could be involved, one has to be aware of its atypical manifestations. We present three uncommon manifestations of the disease. Our first case presented with fever, arthralgias, and right hilar lymphadenopathy with a history of tuberculosis in the past. He was treated for tuberculosis but had a relapse of symptoms three months after completion of treatment. The second patient presented with a headache for two months. On evaluation, cerebrospinal fluid examination showed evidence of aseptic meningitis, while an MRI of the brain demonstrated enhancement of the basal meninges. The third patient was admitted with a mass on the left side of the neck for one year. On evaluation, he was found to have left cervical lymphadenopathy, with its biopsy showing non-caseating epitheloid granuloma. Immunofluorescence did not show evidence of leukemia or lymphoma. All the patients had negative tuberculin skin tests and elevated serum angiotensin-converting enzyme levels supporting the diagnosis of sarcoidosis. They were treated with steroids with complete resolution of symptoms and no recurrence at follow-up. Sarcoidosis is an underdiagnosed entity in India. Thus, awareness of the atypical clinical features could lead to early recognition of the disease and its treatment.

## Introduction

Sarcoidosis is an uncommon multiorgan disease with protean manifestations, characterized histopathologically by non-caseating granulomas in various organ systems [1]. Though it commonly involves the pulmonary parenchyma, lymph nodes, eyes, and skin, it can involve any organ. Thus, the clinical manifestations vary depending on the organs involved. Hence, it features in the differential diagnosis of many clinical conditions. Lack of awareness of the disease, an overlap of its features with that of tuberculosis, absence of any specific diagnostic tests, the cost involved in special investigations, and hence, patient’s reluctance to do them, leads to many patients in India remaining undiagnosed [2] or wrongly diagnosed and treated as tuberculosis. We hereby present three uncommon manifestations of sarcoidosis to create awareness amongst clinicians and facilitate early diagnosis and treatment.

## Case presentation

Case 1

**Figure 1:**
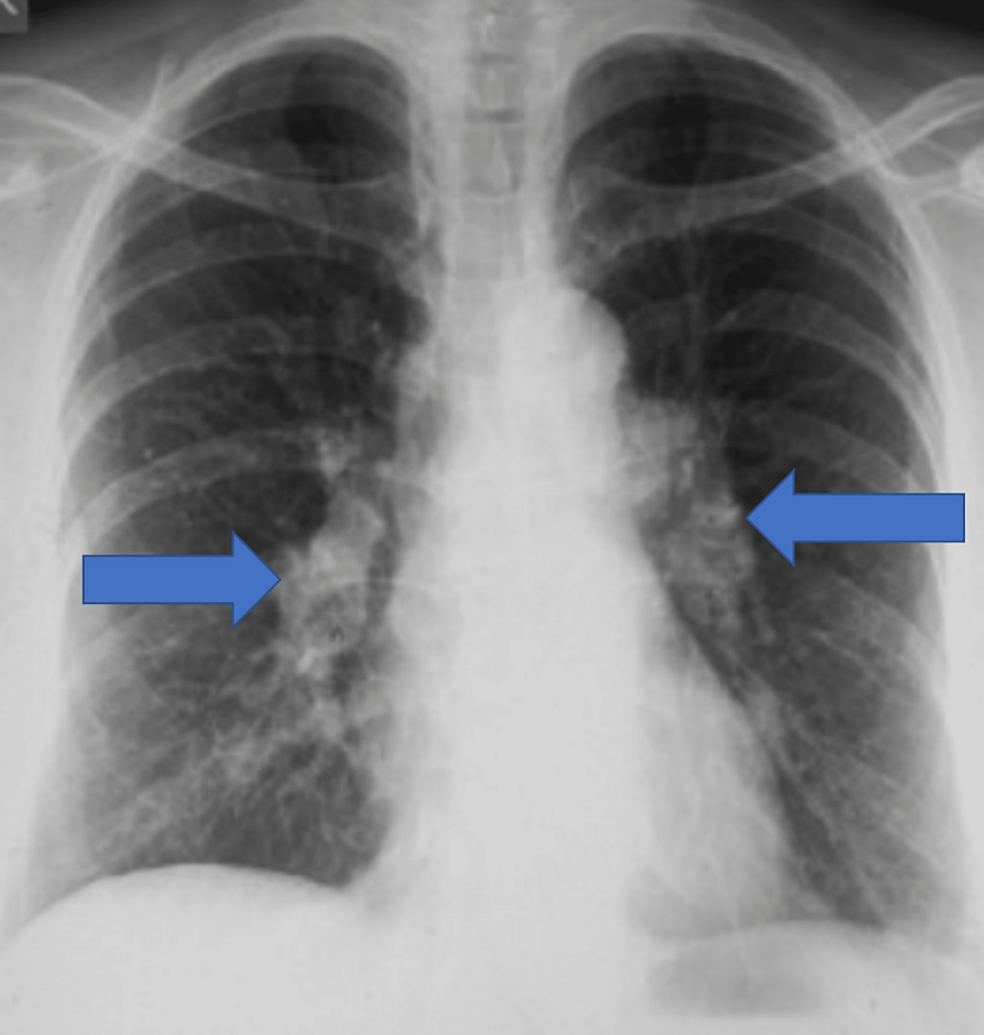
Chest radiograph showing bilateral hilar lymphadenopathy.

A 42-year-old gentleman presented with intermittent fever of six months with joint pains involving small and large joints of the body during the peak of fever. There was no joint swelling or stiffness. He had no earlier co-morbidities. There was a history of tuberculosis 10 years back, for which he had taken treatment for six months. There was no exposure to domesticated pets. On examination, he was coherent; there was no pallor, jaundice, cyanosis, and lymphadenopathy. He was febrile with a temperature of 101 Fahrenheit, pulse rate of 100/minute, blood pressure (BP) of 112/78 mm Hg, and respiratory rate of 21/minute. Systemic examination was within normal limits. His chest radiograph was normal. Contrast-enhanced computerized tomography (CECT) of the chest revealed an enlarged right hilar lymph node of 2.1x1.8 cm and normal lung parenchyma. His detailed evaluation of the cause of the fever did not reveal any specific cause. Given the prolonged history of fever, tuberculosis, enlarged right hilar lymph node, and negative work-up for other causes of fever, he was diagnosed with extra-pulmonary tuberculosis and started empirically on four-drug anti-tubercular therapy (ATT) for six months. He completed ATT without any complications and remained asymptomatic for the next three months, after which there was a relapse of fever with joint pains. This time, suspecting sarcoidosis, his serum calcium (Ca2+), serum angiotensin-converting enzyme (ACE), and vitamin D levels (Table 1) and repeat CECT thorax were done. Given elevated serum ACE levels with bilateral hilar lymphadenopathy (Figure 1), the diagnosis was revised to sarcoidosis, and he was started on oral prednisolone 20 mg/day. This was gradually tapered over six months with complete resolution of symptoms, normalization of serum ACE levels, and resolution of lymph nodes. At four years of follow-up, he remains asymptomatic.

**Table 1:** Significant biochemical investigations of the three patients. TLC: Total leucocyte count; ACE: Angiotensin-converting enzyme.

Parameters	Case 1	Case 2	Case 3	Normal range
Hemoglobin (gm/dL)	10.6	11.7	10.8	11.5-16.5
TLC (cells per mm^3^)	4,900	5,600	6,200	4,000-11,000
Platelet count (cells per mm^3^)	148,000	153,000	142,500	150,000-450,000
Serum 25 OH D_3 _(ng/ml)	18.1	24.3	36.8	20-40
Serum calcium (mg/dl)	8.9	9.8	11.8	8.5-10.2
Serum ACE (mcg/L)	89.7	98.2	126.8	< 40

Case 2

A 24-year-old gentleman was admitted with an intermittent diffuse headache for two months. There was no diurnal variation of headache. It was not associated with fever, vomiting, double vision, blurred vision, photophobia or phonophobia, watering of eyes, nasal congestion, involuntary movements, and giddiness. There was no past or recent trauma to the head. There were no co-morbid conditions and history of exposure to tuberculosis. On examination, he was coherent; there was no pallor, jaundice, cyanosis, or lymphadenopathy. All vital parameters were within normal limits. The central nervous system examination revealed terminal neck stiffness and no focal neurological deficit. Routine blood parameters were within normal limits except for high C-reactive protein (CRP) 5.24 mg/dl (normal: 0.5 to 1.3 mg/dl). The chest radiograph was unremarkable. Non-contrast CT (NCCT) brain was within normal limits. A cerebrospinal fluid (CSF) examination showed 120 cells with 72% lymphocytes, 28% neutrophils, and no abnormal cells. Total CSF proteins were 89 mg/dl, and sugar was 86 mg/dl (corresponding blood sugar was 102 mg/dl). The adenosine deaminase (ADA) level was 5 U/l. Gram-stain did not reveal any organisms, smear for acid-fast organisms was negative, and India ink preparation was negative for cryptococcus. The culture was sterile. The nucleic acid amplification test of CSF (GenXpert) was negative for acid-fast bacillus. MRI of the brain with T2 image showed enhancement of the basal meninges. The rest of the MRI was unremarkable. He was non-reactive for HIV I and II. His CECT thorax and abdomen did not reveal any pathology. Rheumatological tests like rheumatoid factor (RA), anti-cyclic citrullinated antibody (anti-CCP), anti-nuclear antibody (ANA), and anti-double stranded deoxyribonucleic acid (dsDNA) antibody were negative. Serum ACE levels were high (98.2 mcg/L). He was treated with high-dose oral prednisolone given neurosarcoidosis (1mg/kg) for four weeks and then gradually tapered over the next six months. Within two weeks of starting prednisolone, he was free from headache.

Case 3

**Figure 2:**
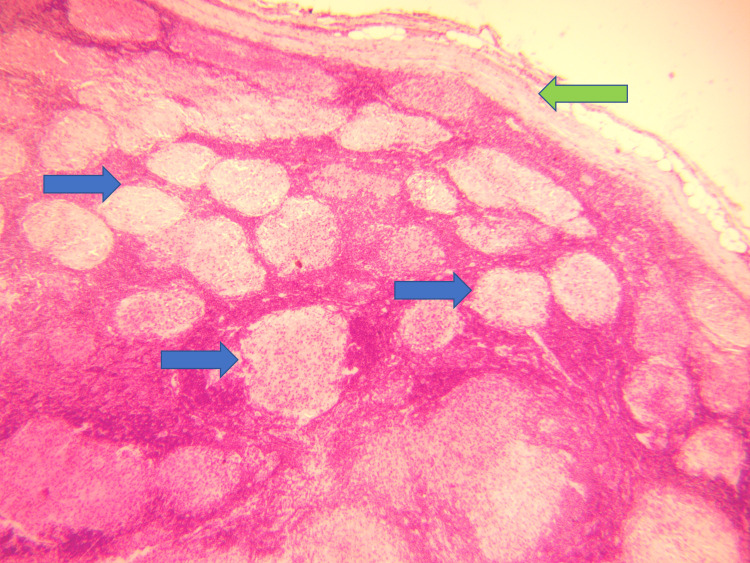
H&E stain (100x) showing lymph node structure with fibrous capsule (green arrow) and well-defined granulomas (blue arrows). H & E Stain: Haematoxylin and Eosin stain

**Figure 3:**
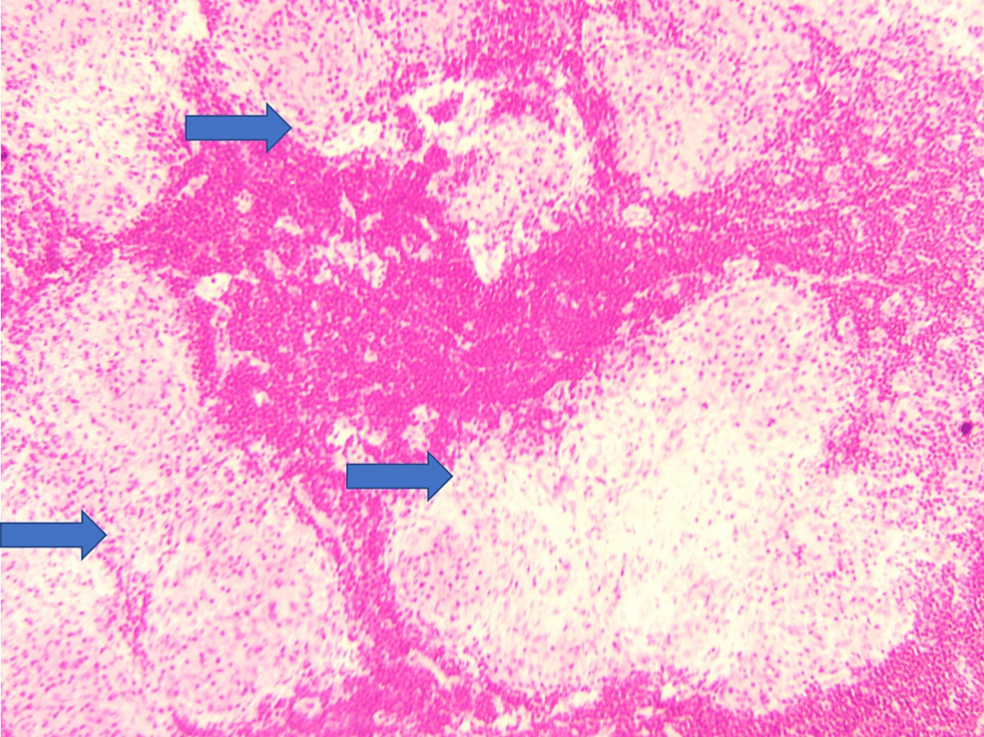
H&E stain (100x) showing well-defined non-caseating granulomas.

**Figure 4:**
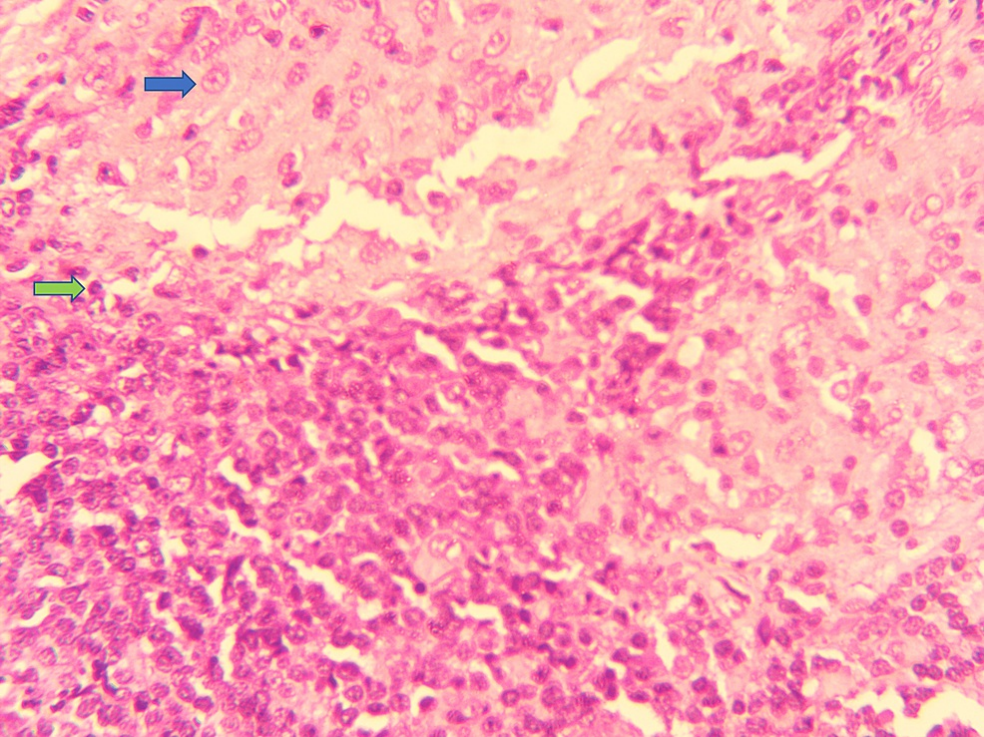
H&E stain (400x) showing well-defined non-caseating granuloma comprised of epithelioid histiocytes (blue arrow) rimmed by mature lymphocytes (green arrow).

**Figure 5:**
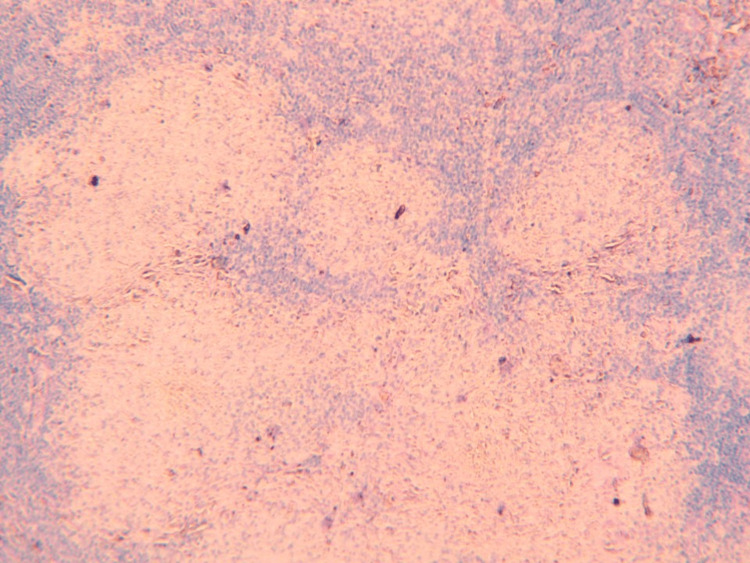
AFB stain (100x) showing negative for acid-fast bacilli. AFB: Acid-fast bacilli.

A 65-year-old male was admitted for swellings on the left side of the neck for one year. The swellings were painless and gradually increasing in size. There was no history of fever, night sweats, or weight loss. The patient had undergone mitral valve replacement (MVR) for rheumatic mitral valve disease four years back and was on oral warfarin 5 mg once daily. There was no other co-morbid condition. On examination, he did not have pallor, jaundice, and cyanosis. There were two discrete, firm, non-tender upper cervical lymph nodes, one measuring 1.5 cm x 2 cm and the other measuring 1.2 cm x 2.5 cm, not fixed to the underlying structures. The overlying skin was pinchable, and there were no inflammatory signs. The vital examination was normal except for an irregular pulse rate of 70-80 beats/minute. Examination of the cardiovascular system revealed normal mitral valve clicks and atrial fibrillation with controlled ventricular rate. The rest of the systemic examination was normal. Routine blood parameters were within normal limits except for a high CRP of 8.24 mg/dl. His serum calcium (Ca2+), serum ACE and vitamin D levels were as in Table 1. A left cervical lymph node biopsy revealed non-caseating granulomas with epitheloid cells morphologically favoring sarcoidosis. There was no evidence of malignancy, lymphoma, or tuberculosis (Figures 2-5). Immunohistochemistry of the lymph node did not reveal evidence of lymphoma or malignancy.

**Figure 6:**
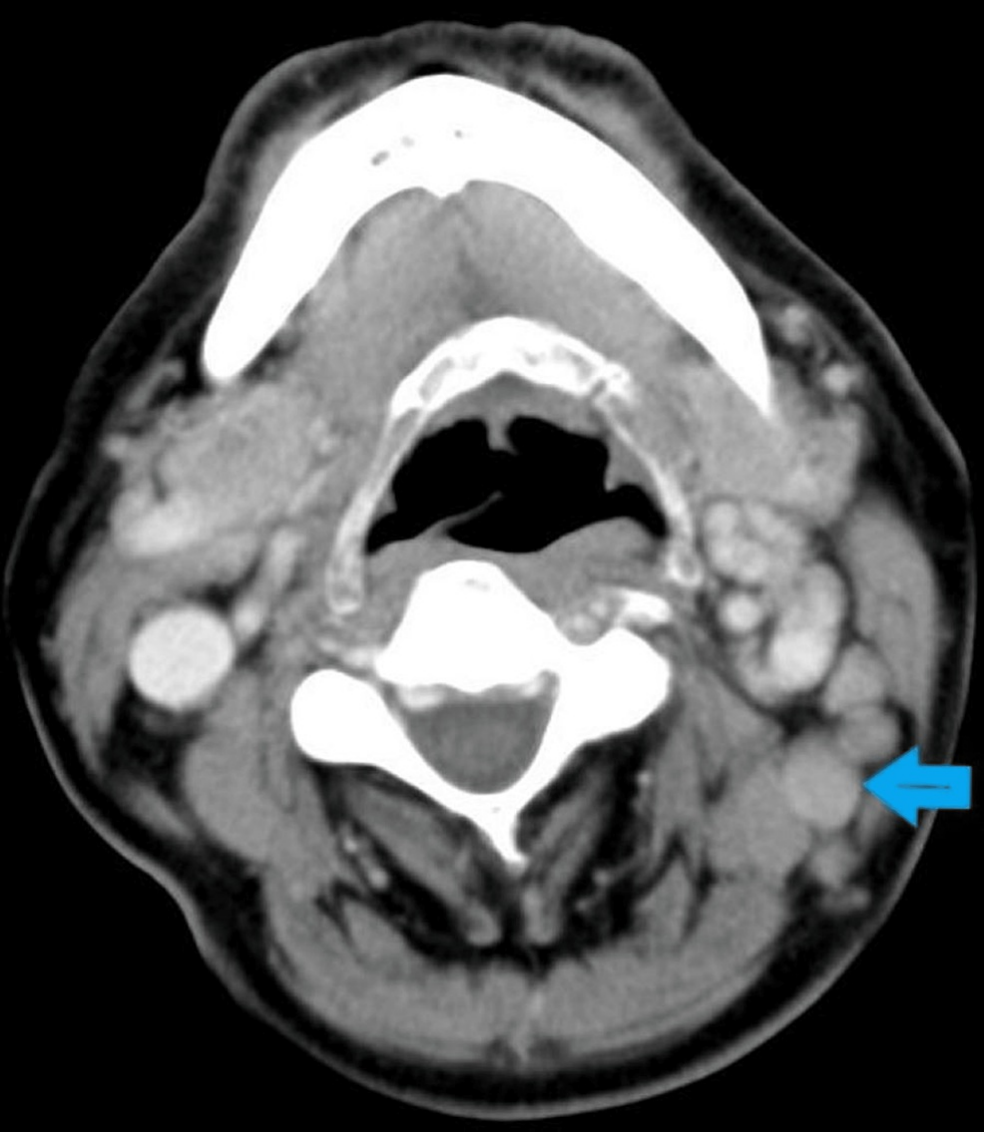
A CECT scan of the neck shows enlarged multiple left jugular lymph nodes. CECT: Contrast-enhanced computed tomography.

CECT neck and thorax revealed enlarged left jugular lymph nodes (Figure 6) and normal lung parenchyma with few small mediastinal lymph nodes, respectively. There was no evidence of calcification or necrosis.

**Figure 7:**
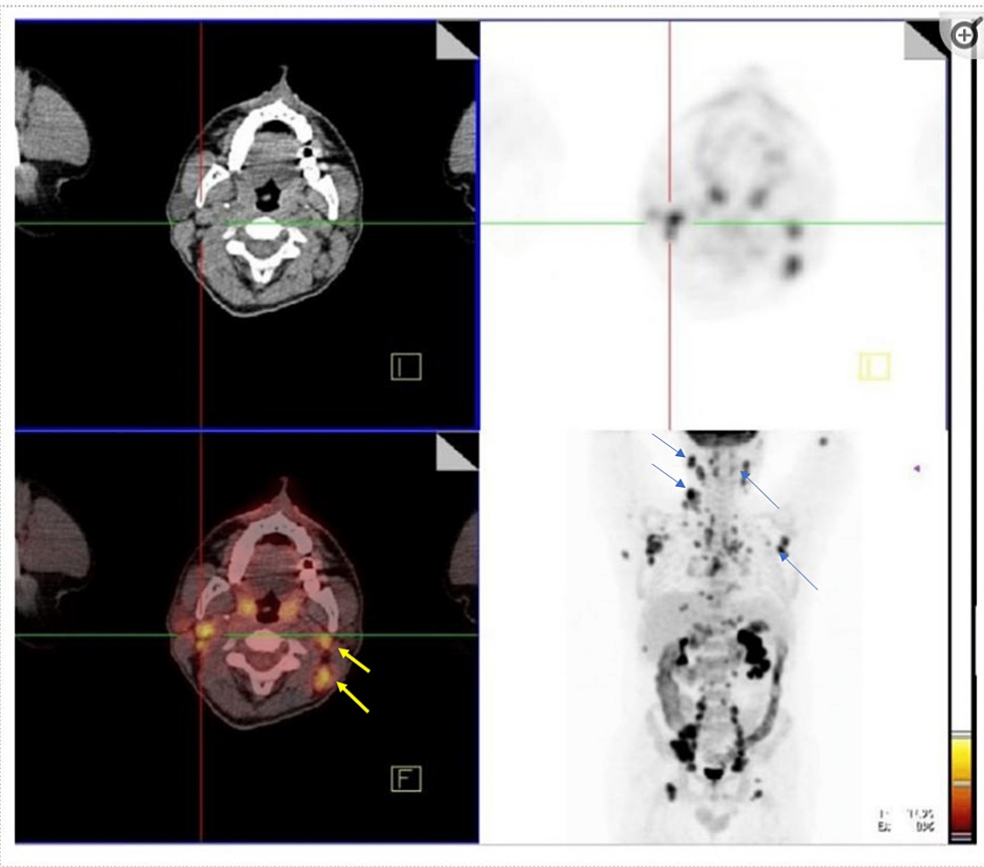
PET scan (whole-body) showing increased metabolic activity in the cervical, mediastinal, retroperitoneal, para-aortic lymph nodes, liver, and spleen. PET: Positron emission tomography.

Positron emission tomography (PET) (Figure 7) of the whole body showed multiple subcentimeter mediastinal lymph nodes with no significant 18F-fluorodeoxyglucose (FDG) uptake and few FDG avid lymph nodes in the left cervical region at levels II-V (standardized uptake - SUV: maximum 9.7), retroperitoneal and para-aortic lymph nodes (SUV: 10.7). 

He was put on 20 mg of oral prednisolone once daily and was instructed to avoid a high-calcium diet. During his subsequent follow-up in the outpatient clinic at four weeks, his serum calcium had normalized to 9.2 mg/dl. All three patients tested negative for a tuberculin skin test.

## Discussion

Sarcoidosis is a systemic granulomatous disease whose etiology is unknown. Certain infectious agents, environmental factors, and associations with human leucocyte antigen (HLA)-B8 and -DR3 have been implicated [3]. It can affect persons of any age, race, and sex but commonly affects young and middle-aged adults. The prevalence of sarcoidosis is about 5 to 50 per 100,000 population, with the highest prevalence reported in Northern Europe [4]. It generally involves the hilar lymph nodes, lung parenchyma, eyes, and skin. Histology is characterized by non-caseating epithelioid cell granulomas in the affected organs. Tumor necrosis factor α (TNF-α) is believed to play an essential role in the proliferation of macrophages and T lymphocytes and granuloma formation [5]. The diagnosis is established based on compatible clinical and radiologic findings supported by histology. Diagnosis, however, is not based on histological findings alone. Sarcoid-like granulomas may be found in conditions other than sarcoidosis, like malignancy, and other granulomatous reactions like tuberculosis, fungal infections, syphilis, berylliosis, lymphoma, and foreign reaction [3]. Clinical features depend upon the organs involved. The typical clinical manifestations include systemic symptoms like fever, body pains, night sweats, weight loss, erythema nodosum, bilateral hilar lymphadenopathy, pulmonary infiltration, and cutaneous and ocular lesions. However, fifty percent of the patients may remain asymptomatic and picked up incidentally by chest radiograph [5]. Our first patient had systemic symptoms with a unilateral right hilar lymph node. However, considering the history of tuberculosis and its prevalence in India, we considered it the first possibility. It was only during the recurrence of the symptoms after three months possibility of sarcoidosis was considered, and it corroborated with increased serum ACE levels. Further, the patient developed bilateral hilar lymphadenopathy characteristic of sarcoidosis and responded to steroids without relapse till four years of follow-up.

The second case was that of a neurosarcoidosis. Neurological involvement in sarcoidosis occurs in 3-10% of the cases [6]. It can affect any part of the nervous system. Involvement of the cranial nerves, basal meninges, and brain parenchyma are common. Cranial mononeuropathy involving the seventh cranial nerve is the most common presenting manifestation though other cranial nerves may also be affected. Mononeuritis multiplex, sensorimotor peripheral neuropathy, aseptic meningitis, hydrocephalous, seizures, and psychosis are the other disease manifestations. About 90% of the patients have systemic manifestations when they present with neurological features [6]. However, neurological features may be the first presenting manifestation in some cases. In a study by Leonhard SE et al. involving 52 cases of probable neurosarcoidosis, neurological manifestations as the presenting symptoms were seen in 71% of the cases [7]. Chronic septic meningitis was the most common manifestation in 37%, followed by cranial mononeuropathy in 31% of the cases. However, in an Indian study on "Uncommon manifestations of Sarcoidosis" by Sharma SK et al. from AIIMS involving 93 confirmed cases, neurological manifestations were observed only in eight patients. They included optic neuritis, optic atrophy, multiple brainstem lesions, proximal myopathy, and pituitary stalk lesion [2]. Noble JM et al. described a case of a 27-year-old man who presented with rapid delirium and profound inflammatory changes in CSF. CSF protein levels were so high that there was a spinal block or Froin's syndrome [8]. Our case presented with chronic aseptic meningitis with raised serum ACE levels without other systemic manifestations. The diagnosis of neurosarcoidosis is challenging as it is difficult to obtain neural tissue for histopathology. Our third patient presented with isolated left cervical lymphadenopathy without systemic symptoms and evidence of sarcoidosis elsewhere clinically. The diagnosis in such cases becomes a challenge. Sarcoidosis accounts for 1.7% of all head and neck lymphadenopathy. In a three-year study, "A case-control etiologic study of sarcoidosis (ACCESS)," conducted by Newman LS et al. involving 736 patients, only six patients had isolated head and neck sarcoidosis [9]. Sarcoidosis is still an under-reported disease in India. It affects all ethnic groups with minor differences in the regional patterns of the disease. Indian studies have shown male preponderance with cases presenting late in the fourth or fifth decade of life. A retrospective study done by Sharma SK et al. on the "Rare manifestations of sarcoidosis in modern era of new diagnostic tools" describes the manifestations in 164 confirmed cases over five years [10]. All our cases had atypical presentations with high serum ACE levels helping in the diagnosis. ACE is produced by epithelioid cells derived from activated macrophages in the granuloma [11]. Its level correlates with the amount of whole-body granuloma and, thus, disease severity. However, ACE levels are also elevated in other granulomatous diseases such as leprosy, histoplasmosis, silicosis, tuberculosis, and non-granulomatous disorders like hyperthyroidism and Hodgkin's lymphoma, thus, limiting their role in the diagnosis of sarcoidosis [11]. One of our patients had mild hypercalcemia. In Western literature, the reported incidence of hypercalcemia in sarcoidosis varies from 2 to 63%. It is related to the dysregulated production of 1,25-(OH)2-D3 (calcitriol) by the activated macrophages in the granuloma [12]. However, as there is no gold-standard test in the diagnosis of sarcoidosis, we relied on serum ACE levels in the context of appropriate clinical settings, response to therapy, and follow-up to make the diagnosis.

## Conclusions

These cases reiterate the myriad clinical presentations of sarcoidosis. It is an inflammatory disorder with multi-system involvement. Hence, it should be included in the differential diagnosis of cervical lymphadenopathy, aseptic meningitis, and unexplained fever with arthralgias. As the disease closely resembles other granulomatous disorders like tuberculosis, it is necessary to rule it out by necessary investigations to avoid getting wrongly treated for the same. Clinicians should be cognizant of the varied clinical manifestations of the disease.
